# Constraint-based analysis of metabolic capacity of *Salmonella typhimurium *during host-pathogen interaction

**DOI:** 10.1186/1752-0509-3-38

**Published:** 2009-04-08

**Authors:** Anu Raghunathan, Jennifer Reed, Sookil Shin, Bernhard Palsson, Simon Daefler

**Affiliations:** 1 Department of Infectious Diseases, Mount Sinai School of Medicine, New York, USA; 2 Department of Chemical and Biological Engineering, University of Wisconsin, Madison, USA; 3 Department of Bioengineering, University of California San Diego, San Diego, USA

## Abstract

**Background:**

Infections with *Salmonella *cause significant morbidity and mortality worldwide. Replication of *Salmonella typhimurium *inside its host cell is a model system for studying the pathogenesis of intracellular bacterial infections. Genome-scale modeling of bacterial metabolic networks provides a powerful tool to identify and analyze pathways required for successful intracellular replication during host-pathogen interaction.

**Results:**

We have developed and validated a genome-scale metabolic network of *Salmonella typhimurium *LT2 (iRR1083). This model accounts for 1,083 genes that encode proteins catalyzing 1,087 unique metabolic and transport reactions in the bacterium. We employed flux balance analysis and *in silico *gene essentiality analysis to investigate growth under a wide range of conditions that mimic *in vitro *and host cell environments. Gene expression profiling of *S. typhimurium *isolated from macrophage cell lines was used to constrain the model to predict metabolic pathways that are likely to be operational during infection.

**Conclusion:**

Our analysis suggests that there is a robust minimal set of metabolic pathways that is required for successful replication of *Salmonella *inside the host cell. This model also serves as platform for the integration of high-throughput data. Its computational power allows identification of networked metabolic pathways and generation of hypotheses about metabolism during infection, which might be used for the rational design of novel antibiotics or vaccine strains.

## Background

*Salmonellae *are Gram-negative bacterial pathogens with a wide host range. The ability of *Salmonella enterica *to cause disease is closely correlated with its ability to survive inside its host cells, which include epithelial cells and macrophages [1]. Infection of mice with *Salmonella enterica *serovar Typhimurium (*S. typhimurium*) serves as model for the systemic disease typhoid fever. *Salmonella *strains that are deficient for intra-macrophage survival cannot elicit disease in the mouse model. Many genomic and proteomic datasets exist for *Salmonella *as a pathogen [2-6]. Most experiments have focused on identifying 'virulence' factors that might drive the disease process, such as surface structures or secreted products. Differential expression of metabolic gene-products has largely been left unexplored in terms of their role in pathogenesis. However, it has now gained more attention that the metabolism of intracellular bacterial pathogens such as *Salmonella *may hold important clues to understanding their intracellular life-style as well as to understanding host defense mechanisms [7,8]. Genome-scale metabolic *in silico models *that can integrate and exploit genomic and proteomic data may provide a first step towards a quantitative analysis of the metabolism of intracellular bacteria.

Constraint-based reconstruction and analysis (COBRA) of cellular metabolism has been employed successfully to develop organism-specific genome-scale *in silico *models across all the three major domains of the tree of life [9-12]. The COBRA approach seeks to differentiate between those network states that are achievable by the organism from those that are not, based on a detailed reconstruction of metabolism and incorporation of physiological parameters that are consistent with known experimental information [13]. The solutions this analysis provides are based on the successive imposition of governing physicochemical constraints on genome-scale reconstructions.

COBRA integrates and represents our current knowledge of network components and interactions and has been applied to several prokaryotic and eukaryotic organisms, including *Escherichia coli *[9], *Helicobacter pylori *[14], *Haemophilus Influenzae *[15], *Saccharomyces cerevisiae *[16] and *Geobacter sulfurreducens *[17]. By systematizing and providing context for transcriptomic, proteomic, and metabolomic data, these models allow for the imposition of constraints, which together define the possible phenotypic behavior allowed by these *in silico *organisms. The use of constrained based modeling has significant potential to identify new antibacterial targets [18]. Modeling allows simulation of single gene knockouts and, more importantly, the effect of combinatorial deletions, which might allow the rational development of combination drug therapy. The use of such a modeling approach has been demonstrated for *H*. *influenza *[15] and for *Mycobacterium tuberculosis *[8]. As the number of pathogens with such models grows [19-24,8], one might expect that more basic principles of bacterial metabolism needed for pathogenesis can be derived.

Here we describe the reconstruction of a detailed and validated genome-scale metabolic network for *S. typhimurium*, that reconciles established genomic and high-throughput phenotyping data and that is augmented by experimental data to facilitate iterative model building efforts. Furthermore, we demonstrate how the model can be used to reconcile existing virulence data and to resolve gaps in our understanding of pathogen metabolism and host-cell nutrient environment. We also use gene expression data obtained for this study in combination with established microarray and proteomic data in the context of the model to understand pathogen metabolism in macrophage cell lines.

## Results

### Characteristics of the Reconstructed Metabolic Network

**Figure 1:**
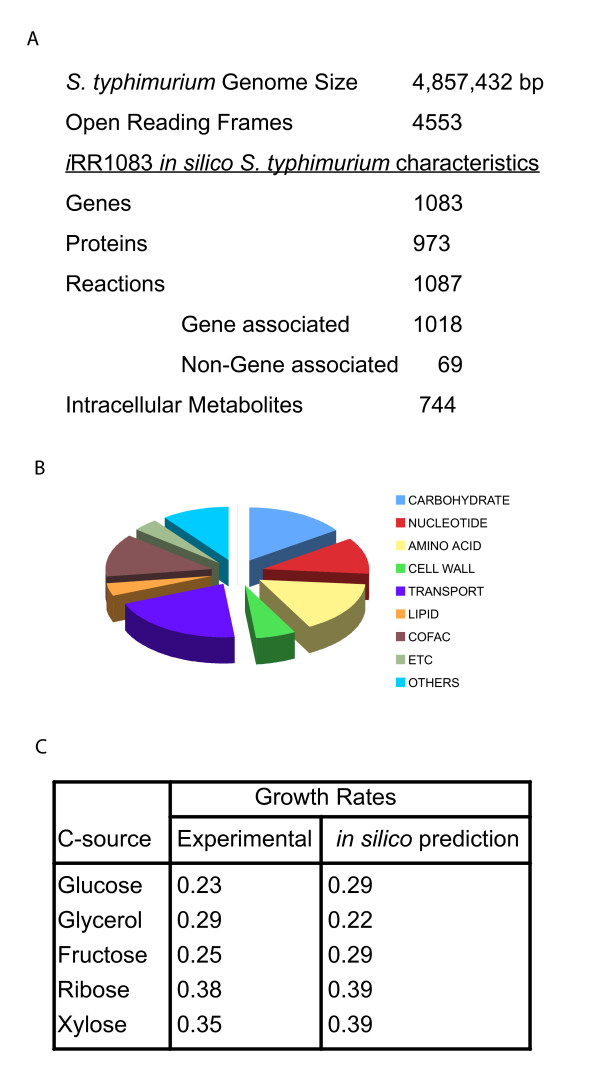
**Characteristics of metabolic network *i*RR1083 for Salmonella typhimurium**. (A) Statistics of the S. typhimurium genome and in silico reconstruction *i*RR1083. The in silico representation includes Gene-Protein-Reaction relationships including isozymes, multimeric protein complexes, and multi-reaction catalyzing enzymes as described in the text. (B) Functional classification of genes included in the model. (C) Comparison of observed growth rates (experimental) with growth rates predicted by model iRR1083 providing different carbon sources under aerobic culture conditions. Experimental growth rates were calculated using the Monod growth equation based on O.D values and a biomass calibration curve as described in Materials and Methods. For predictions, experimental specific substrate uptake rates were calculated from HPLC profiles obtained at different time points in the growth curve.

The 4,857-kilobase (kb) chromosome of *Salmonella enterica *serovar *typhimurium *LT2 (*S. typhimurium LT2*) accounts for 4,489 coding sequences (CDS/ORFs) including 39 pseudogenes and a 94 kb plasmid encoding 108 ORFs [25]. The *in silico *metabolic reconstruction of the bacterium referred to as *i*RR1083 contains only annotated chromosomal genes. The characteristics of the network are detailed in Figure 1A and 1B (for details see Additional file 1). It currently accounts for 1,083 genes that encode proteins catalyzing 1,087 unique metabolic and transport reactions in the bacterium. The reconstruction includes gene to protein to reaction association relationships, which distinguish between isozymes, multimeric proteins and protein complexes [26]. The reconstruction contains all the biosynthesis pathways to make biomass components that are required for growth. A biomass production reaction was formulated, which is based on the measured biomass composition of the bacterium and which details the required metabolites and their relative amounts.

The reconstruction accounts for central metabolism, carbohydrate utilization, amino acid biosynthesis and degradation, nucleotide biosynthesis and salvage, and cofactor biosynthesis (see Additional file 1). Also included were anaerobic and aerobic electron transport chain reactions, transport reactions for various minerals and small molecules, and biosynthesis reactions for cell wall constituents and precursors, such as phospholipids and peptidoglycan. Lipid biosynthesis in bacteria involves (i) fatty acid biosynthesis, (ii) attachment of the complete fatty acids to sn-glycerol-3-phosphate, and (iii) addition and modification of head groups to yield the major cellular phospholipids. Fatty acids are important constituents of lipid A and the cell wall of bacteria. In contrast to published models of other microbes that used compositional data of *E. coli*, we have directly measured amino acid, fatty acid and lipid profiles for *Salmonella *LT2 (see Additional file 2) and incorporated them into our reconstruction in *i*RR1083. Since fatty acid synthesis has also become a recent target of new antimicrobial drugs, it is critical to represent fatty acids and lipids accurately for different pathogens.

In addition to accounting for these major aspects of cell metabolism and biosynthesis, the reconstruction also includes reactions required for drug efflux, proton pumps, and inhibitory effects of antibiotics on bacterial metabolism. We have included the AcrAB-TolC multi-component drug efflux pump which has been characterized in *S. typhimurium*. This efflux pump has been shown to be involved in the resistance to β-lactams (ampicillin and penicillin G), fluoroquinolone (ciprofloxacin and fluoroquinolone), cefotaxime, and chloramphenicol [27-29]. An increase in the expression levels of this pump has been recognized as a significant determinant of antibiotic resistance in bacterial pathogens. The inclusion of this efflux pump allows modeling of the reaction of *Salmonella *to antibiotics, by varying efflux pump activity and/or limiting metabolic flux through the respective pathways. For example, β-lactam antibiotics result in incomplete peptidoglycan synthesis (D-Ala-D-Ala addition), which can be modeled by restricting flux through peptidoglycan synthesis. Similarly, dihydropteroate synthase inhibition by sulfonamides and dihydrofolate reductase inhibition by trimethoprim can be simulated by constraining fluxes through their corresponding reactions.

Another unique aspect of our reconstruction are the reactions involved in free radical quenching that are generally used by *Salmonella *to counteract the host cell response to infection [30,31]. In addition to the classically known mechanisms mediated by superoxide dismutases, catalases and the enzymes that make and recycle glutathione and thioredoxin, our reconstruction includes mechanisms for reactive nitrogen species (RNS) and reactive oxygen species (ROS) resistance and removal. Some of those include: a soluble flavohemoglobin (Hmp) with a NO dioxygenase activity that is induced solely by RNS; and an alkyl hydroperoxide reductase (AhpC) belonging to the peroxiredoxin family that is involved in dissipating both ROS and RNS [31-34]. The inclusion of reactions for ROS (superoxide, peroxide) and RNS (nitrate, nitrite, nitric oxide, and nitrous oxide) species make this model a suitable platform to analyze their role in infection and pathogenesis.

### Growth predictions

Flux balance analysis (FBA) was used to predict growth rates on six different carbon sources (glucose, fructose, arabinose, xylose, ribose and glycerol) using their measured substrate uptake rates. These *in silico *predicted growth rates were then compared to experimental results of aerobic batch cultivation of *S. typhimurium *LT2 in M9 minimal media. The calculated maximum specific growth rate (hr^-1^) of *Salmonella *for a given carbon source uptake rate was close to the experimentally determined values for *S. typhimurium *under similar conditions (Figure 1C). Quantitative differences between calculated and observed growth rates could be attributed to differences in ATP maintenance parameters (values for *E. coli *were used in the simulations [9]) or secretion of by-products, which were not predicted in any of the simulations since carbon sources were assumed to be the limiting nutrient.

*S. typhimurium *is a mixed acid fermenter that metabolizes glucose via the Embden-Meyerhoff-Parnas pathway [35]. Many of the predicted non-zero fluxes (as calculated using FBA) corresponded to genes whose mRNA transcript expression levels were measurable during growth of *Salmonella *(see Additional file 8) on glucose minimal media. Most of the transcripts detected in our study concur with previous reports in literature. During growth on glucose the iRR1083 model predicts that oxaloacetate can be produced by phosphoenolpyruvate carboxylase (PepC), which is known to be the only enzyme serving the anaplerotic role of replenishing oxaloacetate that is withdrawn for amino acid biosynthesis. In agreement with our flux calculations, our experimental results also indicate a high abundance of the PepC mRNA. The data also indicate that glucose is converted to biomass and some part of the carbon is diverted into stoichiometric formation of formate, acetate, ethanol and succinate under anaerobic conditions [35].

Growth of *S. typhimurium *on 3 pentoses (D-arabinose, D-ribose and D-xylose) was also tested experimentally. Growth was only observed on D-xylose and D-ribose, which is consistent with literature and model predictions [36]. Xylose is catabolized, as most other aldopentoses, by transport into the cell, isomerization and phosphorylation of ketopentose (xylulose). The phosphorylated derivative is then converted to xylulose-5-phosphate by an epimerase and channeled into the pentose phosphate pathway. D-ribose is transported into the cell, concomitant with the disruption of a high-energy phosphate bond and enters the pentose phosphate pathway following epimerization. These results highlight the utility of using modeling to predict growth on substrates *a priori *to experimentation and can help understand the different pathways that are operational during growth on specific carbon and nitrogen sources.

### Qualitative Growth Phenotypes

FBA was also used to predict which of the 163 carbon containing compounds and 98 nitrogen-containing compounds, with transport reactions (excluding antibiotics) that are included in the model, could be used for growth as sole carbon or nitrogen sources. The *in silico *predictions were then compared to (1) existing high-throughput phenotyping data from Biolog's Phenotyping MicroArray™ technology (Biolog Hayward, Inc., CA; data was available from the company website) and (2) reports from experimental literature [37]; (Table 1, see Additional file 4 for complete details).

**Table 1:** *In silico *predictions and experimental data.

**Model Predictions**	Experimental Growth	Experimental No Growth	No Data	Total
**Growth (Carbon)**	75	21	17	113
**No Growth (Carbon)**	1	28	21	50
**Growth (Nitrogen)**	37	5	8	50
**No Growth (Nitrogen)**	9	23	16	48

Comparisons between model predictions (rows) and experimental data (columns) taken from Biolog and published literature

The model predicted that 113 out of 163 potential carbon sources could support growth as the sole carbon source, and 50 out of 98 potential nitrogen sources could support growth as the sole nitrogen source. Comparisons with experimental data could be made for 125 of the potential carbon sources and for 74 of the potential nitrogen sources. Overall, the model predicted carbon and nitrogen sources with 82% accuracy for carbon sources and 81% accuracy for nitrogen sources. The relationship between model predictions and experimental growth phenotypes is statistically significant (chi-squared test statistic yields p < 0.0001 for both carbon and nitrogen sources). Most of the inaccurately predicted carbon sources are compounds that can also serve as sole nitrogen sources experimentally (13 of 21). This suggests that regulation or enzyme kinetic limitations likely prevent these compounds from being used by *S. typhimurium *LT2 as sole carbon sources.

*S. typhimurium *and *E. coli *are closely related microorganisms. Comparison of their physiological data and metabolic network structures suggest differences in carbon and nitrogen source utilization. For example, *E. coli *can utilize D-alanine, L-asparagine, L-aspartate, L-glutamine, and D-lactate as sole carbon sources experimentally, while *S. typhimurium *can not, even though *S. typhimurium *has the necessary enzymes and transporters. These phenotypic differences are likely to be caused by differences in how the organisms control metabolism through regulation and kinetics rather than by differences in metabolic network structure. The same explanation can be made for carbon sources that only *S. typhimurium *can use even though both organisms can catalyze the required reactions (1,2-propanediol, citrate, galactitol, L-glutamate, L-proline, and L-tartarate).*S. typhimurium *is also predicted to be able to utilize additional carbon sources due to the presence of unique transporters and/or enzymes (4-hydroxyphenylacetate, deoxyribose, ethanolamine, myo-inositol, L-lyxose, D-tartrate, D-glycerate, 2-phosphate-D-glycerate, 3-Phospho-D-glycerate, L-cysteine, isocitrate, phosphoenolpyruvate and tricarballylate).

### Analysis of gene deletions, essentiality and lethal genes

The impact of single gene deletions on growth of *S. typhimurium *LT2 was analyzed by simulating its growth on rich medium in the absence of the reactions catalyzed by the corresponding gene. The results were compared for consistency with a compiled list of 255 essential genes from a large-scale gene essentiality screen. Of the 255 essential genes, only 74 were included in iRR1083 and could be compared with experimental results. The total accuracy of essentiality predictions was around 40% (30 out of 74). The accuracy of predicting in vivo essential genes for growth in rich media is within the range reported for models of *Pseudomonas aeroginosa *(40%) [21], *H. pylori *(57%)[14], and *Bacillus subtilis *(69%)[11]. Discrepancies between model predictions and experimental results can be used to better identify what metabolites are present in the rich media and lead to model improvements. For example, inclusion of charged tRNAs rather than free amino acids into the biomass reaction would lead to the correct prediction of amino acid tRNA synthetases as essential genes.

To further explore the metabolic differences between *E. coli *and *S. typhimurium *we compared gene essentiality predictions for *in silico *aerobic growth in glucose minimal media using the most recent *E. coli *metabolic reconstruction, *i*AF1261 [9] and *i*RR1083 (see Additional file 5). The number of predicted essential genes was fairly similar for the two organisms, with *E. coli *having 237 (out of 1261) essential metabolic genes and *S. typhimurium *having 200 (out of 1083) essential metabolic genes. The two models have 900 overlapping orthologs, meaning there are 183 genes included in *i*RR1083 which do not have an ortholog included in *i*AF1261 (142 of these do not have orthologs in *E. coli *and another 41 of the *E. coli *orthologs were not included in *i*AF1261).

For 667 of the 900 overlapping orthologs both models predict the genes to be non-essential for *in silico *growth in a glucose minimal medium under aerobic conditions. The models also agree on 136 of the 900 orthologs being essential. Comparing the predictions to experimental *E. coli *knock-out mutant phenotypes (in rich and glucose minimal media [38,39], the conserved non-essential predictions and essential predictions were both 89% accurate (595/667 and 122/136, respectively).

The discrepancies between essentiality predictions made by the two models can be useful in identifying model differences (biomass formulation and reaction reversibility) and metabolic network differences (isozymes and unique reactions) for the two microorganisms. In 45 of the 900 overlapping orthologs, the *E. coli *model predicted genes as essential and the S. typhimurium model predicted those genes as non-essential; in 52 cases the *S. typhimurium *model predicted genes as essential and the *E. coli *model predicted those as non-essential. Most of these discrepancies (~74%) can be attributed to differences in the biomass formulations that were used for the two models. The *S. typhimurium *model either has additional compounds (such as a complete lipopolysaccharide, glycogen, and spermidine) that cannot be produced in a gene's absence (contributing to 35 out of 52 discrepancies), or the *E. coli *model has additional compounds (such as thiamine diphosphate, pyridoxine 5'-phosphate, ubiquinone, and protoheme) that cannot be produced in a gene's absence (contributing to 37 out of 45 discrepancies).

Reaction reversibility differences are also responsible for some of the discrepancies. In the *S. typhimurium *model threonine aldolase is reversible so that threonine can be produced from glycine and acetaldehyde; however, this reaction was changed to irreversible in the updated *E. coli *model, *i*AF1261. As a result the biosynthetic genes for threonine production encoding homoserine kinase (*thrB*) and threonine synthase (*thrC*) are predicted to be essential by the *E. coli *model. In contrast, the *S. typhimurium *model predicts that in their absence threonine can be synthesized from glycine and acetaldehyde. Since both *thrB *and *thrC *are essential for *E. coli *growth in minimal media conditions [38] it is likely that threonine aldolase does not produce threonine. Glycine hydroxymethyltransferase also differs in its reversibility between the two models, where it is reversible in *i*AF1261 and irreversible in *i*RR1083. The reversible reaction provides another pathway in the *E. coli *model for serine production in the absence of phosphoserine phosphatase (*serA*) or 3-phosphoglycerate dehydrogenase (*serB*), and explains why these two enzymes are predicted to be essential for *S. typhimurium *growth and non-essential for *E. coli *growth. Since both *serA *and *serB *are essential experimentally in *E. coli *[38], it is likely that neither organism uses glycine hydroxymethyltransferase for serine production, which is consistent with the *S. typhimurium *model predictions.

Gene essentiality discrepanices caused by metabolic network differences, such as additional reactions and/or isozymes, provide interesting insights. For example, *S. typhimurium *has genes encoding for two separate 3-isopropylmalate dehydratases (encoded by STM0110-STM0111 and STM0329-STM0330) while *E. coli *only has genes for one (encoded by b0071-b0072). As a result, when STM0110 or STM0111 are deleted the *i*RR1083 model predicts that STM0329 and STM0330 can be used instead to produce leucine, which is not possible in *E. coli *since it lacks the second isozyme. *S. typhimurium *also carries out reactions that *E. coli *can not. For example, an NADPH dependent sulfite reductase (unique to *S. typhimurium*) is predicted to be used for hydrogen sulfide production (needed for cysteine biosynthesis) when the NADH dependent sulfite reductase (present in both organisms) is absent.

There are also cases where *E. coli *has additional isozymes or metabolic reactions, that explain why genes are predicted to be non-essential in *E. coli *but essential in *S. typhimurium*. For example, there are multiple carbonic anhydrases and dihydrofolate reductases in *E. coli *while *S. typhimurium *has only one copy of each of these essential enzymes. *E. coli *also contains a gene encoding ribose 1,5-bisphosphokinase, for which there is no ortholog in *S. typhimurium*. This *E. coli *specific enzyme is predicted by the iAF1261 model to compensate for ribose-phosphate pyrophosphokinase in 5-phospho-alpha-D-ribose 1-diphosphate production, which is needed for histidine and nucleotide biosynthesis in the absence of ribose-phosphate pyrophosphokinase [40]. As shown here, comparisons between gene essentiality predictions can be useful in identifying model discrepancies which can lead to updated and improved models, as well as identification of unique metabolic features, which allow for discovery of possible phenotypic differences between two organisms.

### Metabolic gene essentiality for virulence

**Figure 2:**
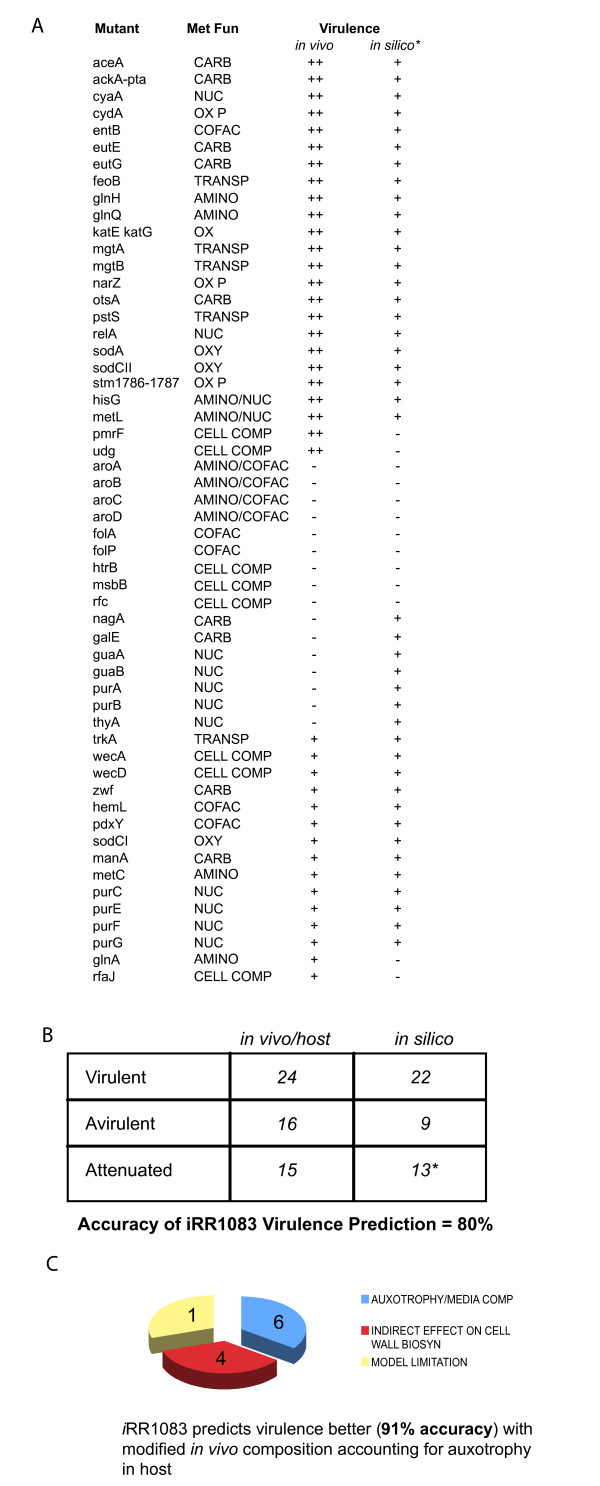
**Gene essentiality Comparison (A) in silico predictions of lethal genes were compared with an experimentally generated screen (in vitro in LB media) that defines an essential metabolic genes set for *S. typhimurium***. ++,-,† and indicates virulent, avirulent, and attenuated mutants. * The model was not used to distinguish between attenuated and virulent phenotypes as this would require detailed nutrient uptake rate measurements. Instead mutants were only predicted to be virulent (maximum growth rate is greater than zero) or avirulent (maximum growth rate is zero). (B) Comparison of in-silico predicted viability and virulence of metabolic gene mutants that have been implicated *in vivo *for virulence. (C) In silico prediction statistics for viability and virulence of the *in vivo *tested mutants (2A) before and after accounting for auxotrophic requirements and nutrient availability in the host.

The identification of genes essential for survival is also important for understanding the minimal requirements for pathogen survival in the host cell and to subsequently identify targets for new antibiotics. A list of genes predicted to be essential for *in silico *growth of *i*RR1083 was compiled and compared with a set of in vivo essential and non-essential genes from the literature (Figure 2A and 2B). Genes essential in vivo are those that are reported to be required for growth. Hence, *Salmonella *mutants defective in these essential genes are avirulent in macrophage or animal models, while mutants defective in non-essential genes are still virulent and can grow. An attenuated mutant strain is capable of growth in vivo at a lower rate than the wildtype. This is consistent with the physiological definition of virulent, avirulent and attenuated strains in literature [41]. Since the availability of nutrients in the macrophage or animal host has not been completely delineated we have used an approximate representation based on that indirectly inferred from existing literature (see Additional file 6). Model predicted growth rates are highly sensitive to nutrient uptake rates which are unknown in the host celll. The model was not used to distinguish between attenuated and virulent phenotypes as this would require detailed nutrient uptake rate measurements. Instead mutants were only predicted to be virulent (maximum growth rate is greater than zero) or avirulent (maximum growth rate is zero).

The *i*RR1083 reconstruction as described earlier only contains metabolic genes, so there are additional non-metabolic essential genes *in vivo *that were not included in our virulence prediction study. The *in silico *virulence predictions matched *in vivo *virulence characterization for 92% (22/24) of the *in vivo *virulent mutants and 56% (9/16) of the *in vivo *avirulent mutants. Most attenuated strains were also correctly predicted by the model to still be able to grow (13/15). Again a statistically significant relationship between model predictions and experimental observations was found (χ^2 ^= 10.9 and p = 0.001). The model predicted incorrectly that mutants defective in *pmrF *and *udg *were avirulent [42,43]. Both of these genes are involved in the biosynthesis of the aminoarabinose component of lipopolysaccharide (which is attached to Lipid A). However, this aminoarabinose component of the LPS is not always present. If alternate LPS structures were also included in the model then the model would accurately predict the virulence of *pmrF *and *udg *mutant strains. This would increase the final virulence prediction accuracy to 100%. The consistencies and discrepancies between *in silico *and *in vivo *mutant phenotypes deserve further discussion and may generate potential hypotheses that can subsequently be tested.

Some genes that are involved in major central metabolic pathways and are non-essential for intracellular survival (i.e. mutants defective in these genes are virulent) are discussed below. *AceA *encodes isocitrate lyase, which catalyzes the first step in the glyoxylate shunt, an anaplerotic carbon assimilatory pathway that synthesizes C4 dicarboxylates from C2 compounds like acetate or short chain fatty acids. AceA is required only for chronic *Salmonella *infections and not for acute infections [44]. This enzyme is generally used when substrates such as glucose or pyruvate are absent and acetate or fatty acids are present. AckA and Pta are two enzymes in salmonella that lead to acetyl phosphate formation and the double deletion strain is virulent. Acetyl-phosphate is thought to activate expression of invasion genes, which can also be activated via a sensory kinase BarA. However, virulence is decreased only when Pta, AckA, and BarA are deleted [45].

Phagocytic cells are able to generate superoxide, hydrogen peroxide and other reactive oxygen species (ROS) and pathogens possess multiple defenses to such oxidative stresses. The model also accurately predicted that catalase (*katE *and *katG*) mutants [46] and super oxide dismutase mutants (*sodA *and *sodCII*) [47,48] are virulent. Mutants lacking a third superoxide dismutase (*sodCI*) are attenuated and the model incorrectly predicts that a *sodCI *mutant would be virulent; however, if the host-cell environment included superoxide and none of the other superoxide dismutases were expressed or active (*sodA*, *sodB*, and *sodCII*) then the bacterium would have no way of dissipating superoxide and the model would predict an avirulent phenotype.

One of the incorrect predictions for the experimentally determined avirulent strains was for the *nagA *mutant. NagA is a N-acetylglucosamine-6-phosphate deacetylase, and it's substrate acts as an inducer of the nag operon, which relieves repression by NagC. It has been proposed that *nagA *mutants indirectly lead to reduction in aminosugar biosynthesis, which is needed in membrane structures [49]. The model's inability to identify this mutant correctly as being avirulent could be due to the absence of any regulatory mechanisms not accounted for in the model. Inclusion of simple regulatory rules as has been done for the *E. coli *model, would allow us to address the role of regulation of metabolism during *Salmonella*-host interaction.

Of the experimentally determined attenuated mutants, the model predicted that mutants defective in aromatic amino acid biosynthesis are avirulent (*aroABCD*). Aro *Salmonella *strains have been used as oral vaccines [50]. The attenuation of this strain, which is accomplished by interrupting pathways for biosynthesis of aromatic amino acids, is consistent with our *in silico *analysis. Strains of *S. typhimurium *with complete non-reverting blocks in aromatic biosynthesis are avirulent [51] as predicted by our model.

**Figure 3:**
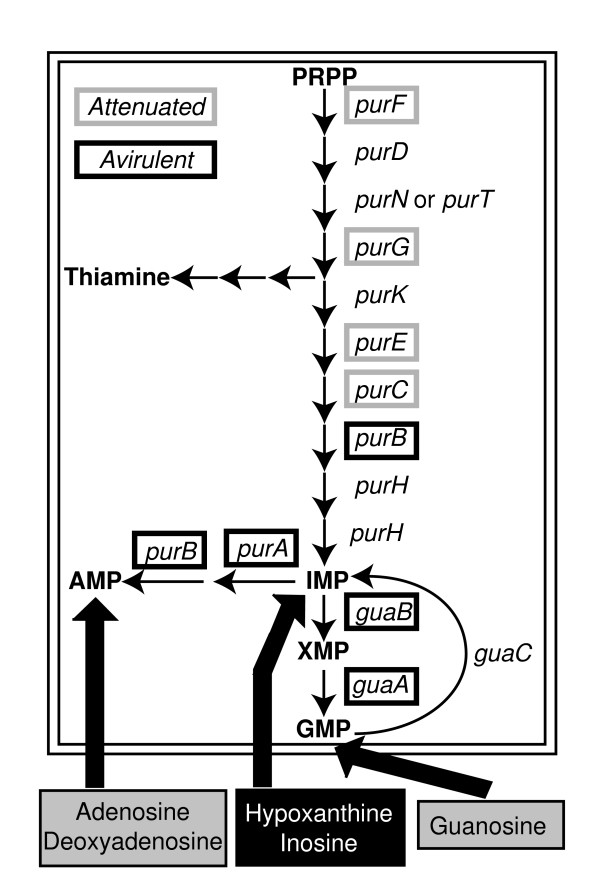
**Attenuated and avirulent mutants defective in purine biosynthesis**. Attenuated mutants are framed in grey and avirulent mutants are framed in black For unframed genes we could not find information about their corresponding mutant phenotypes in the literature. As shown at the bottom, the host cell is assumed to provide purine nucleosides and bases that can be converted into the required nucleotides via salvage pathways. However, *in silico *analysis of mutant phenotypes suggests that not all nucleosides (corresponding to the grey boxes: adenosine, deoxyadenosine and guanosine) are provided in sufficient quantities to allow for auxotrophic growth.

A large portion of the experimentally determined avirulent mutants which were incorrectly predicted by the model to be virulent are associated with nucleotide biosynthesis: *guaAB*, *purAB*, and *thyA *(Figure 3). These mutants were all predicted to be virulent due to the inclusion of thymidine, guanosine, adenosine, and deoxyadenosine into simulations of the host-cell environment. If these metabolites are not available, then the model correctly predicts that these strains are avirulent. Other mutant strains defective in purine biosynthesis genes (*purC, purE, purF*, and *purG*) are attenuated rather than avirulent [51]. The experimental results indicate that intermediates in the purine biosynthesis pathway (eg. hypoxanthine or inosine) are available in the host-cell environment and that guanosine and adenosine are likely produced from these pathway intermediates rather than provided by the host (Figure 3). Better accounting for nutrient availability *in vivo *is then able to resolve 62.5% of the mutants that were incorrectly predicted *in silico *to be virulent. This approach can thus also serve as an indirect probe of the host cell intracellular environment in which the pathogen finds itself.

### Pseudogenes and their role in infection among serovars of *Salmonella*

Sixty-five genes that are included in iRR1083, and thus present in *Salmonella *serovar *typhimurium*, are missing or are pseudogenes in other *Salmonella *serovars (see Additional file 7) [52]. This implies that these genes could be dispensable for virulence. Based on the *in silico *analyses of the effect of single gene deletions on growth of *S. typhimurium *in both rich media and host-cell environments, all 65 genes are predicted to be dispensable. Only one gene out of these 65, *panD*, was predicted to be required for growth in minimal media. PanD is required for panthothenate synthesis, an intermediate in the biosynthesis of CoA. In *E. coli *this enzyme is not required for growth in LB but is required for growth in minimal media [38,39]. Presumably then, serovars lacking *panD *would require panthothenate from its host. Compounds that affect pantothenate transport could potentially be used as antibiotics.

### *Salmonella *Metabolism in infection

**Figure 4:**
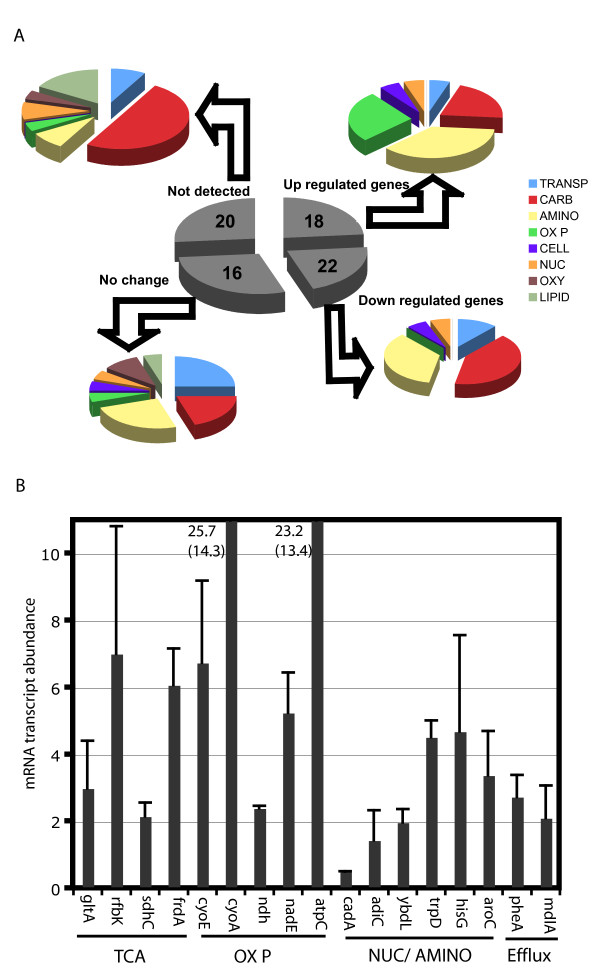
***S. typhimurium *metabolism during infection**. (A) Functional Classification of metabolic gene subsets analyzed for expression in macrophage 24 hr post infection. (B) Subset of metabolic genes up-regulated in *S. typhimurum *24 hr after infection of macrophage cell lines (RAW 246.7). Genes implicated in energy metabolism, heme and aromatic amino acid are most up-regulated.

The transcriptional status of *in vivo *derived bacteria may correlate with mechanisms of pathogen survival and proliferation within host cells. Such genome-scale expression profiles are indicative of the host cell environment as well as the pathogen's adaptation to the intracellular niche. Based on a complete transcriptional profile of intracellular *Salmonella *following short-term (4 h–12 h) infection of macrophage cell lines, only 21% of the total genes in the genome are differentially expressed (Figure 4A). Further, only about 6% of these are characterized metabolic genes of known function. Since it is known that some metabolic genes are essential for intracellular replication [52,53] and that *Salmonella*'s niche in the host has limited nutrient potential, the pathogen appears to have efficiently adapted to minimize its metabolic capacity in order to survive and grow in the phagosome.

We sought to identify genes (Figure 4B) and select pathways that may be crucial for *Salmonella's *infection of and growth inside macrophage cell lines by quantitatively monitoring the expression of 100 selected metabolic genes (see Additional file 8) that are involved in different key pathways. Since only a small percentage of metabolic genes were hypothesized to change mRNA abundance levels during infection, we use the Genomelab Expression profiler (GEXP) for estimating gene expression quantitatively. This technique combines RT-PCR and capillary gel electrophoresis in a high-throughput format to obtain accurate, consistent mRNA abundance levels for expressed transcripts of chosen genes rapidly. Consistent with metabolic gene essentiality hypothesis suggested in literature, only 24% of the genes chosen in this study were up-regulated 24 hrs post infection. Our data indicates that isocitrate lyase (*aceA*), and fatty acid degradation genes *fadA, fadD/fadE *are not expressed. Although these genes have been implicated for virulence in chronic *Salmonella *infection, they are known to be unchanged during short-term infection experiments [2]. Our results suggest the presence of glucose or pyruvate and not acetate or short chain fatty acids *in vivo *during the early stages of infection as suggested in literature [44]. This also explains why genes from the oxidative branch of the TCA cycle (*gltA*, *sdh*) are over expressed.

The simultaneous up regulation of succinate dehydrogenase and electron transport chain genes *cyoE *and *cyoA *is suggestive of an aerobic environment *in vivo*, at least during the early stages of infection [54]. Increased levels of F_1_F_0 _ATP synthase subunits measured within our data set also support this. ATP synthase is primarily responsible for the oxidative phosphorylation of ADP to ATP. It also plays a role in maintaining the proton gradient to maintain intracellular pH and drive efflux pumps. The high levels of mRNA transcripts for succinate dehydrogenase and protoheme farnesyl transferase genes also suggest heme biosynthesis occurs in *S. typhimurium *during infection. These are in concurrence with other existing data sets [5].

Genes belonging to the histidine biosynthesis cluster (*hisC*, *hisD*, *hisG*) were up regulated and those involved in aromatic amino acid synthesis pathways (*aroA*, *pheA*) were down regulated. These findings are consistent with the literature [2]. Genes involved in nucleotide (*purF*) and NAD biosynthesis (*nadE*) were down regulated or not detected. The down regulated genes also included those involved in glycolysis and carbon metabolite repression, certain amino acid decarboxylases and some transporter genes including drug efflux genes. These results suggest that *Salmonella *acquires a significant amount of essential nutrients directly from the host cell, thus shutting down several biosynthetic pathways when inside the host. The genes that cannot be detected include some genes involved in glycolysis/gluconeogenesis (*glk*, *pps*) and anaplerotic reactions (*ppc*). Other genes are detectable, but do not change in expression level over the course of infection, including those in the pentose phosphate pathway (*zwf*), Entner Duoduroff pathway (*edd*) and glutathione cycle (*ggt*, *gst*). Fatty acid biosynthesis and degradation genes are also not detected during the monitored early stages of infection.

### Metabolic capacity of *S. typhimurium *in the host cell

**Figure 5:**
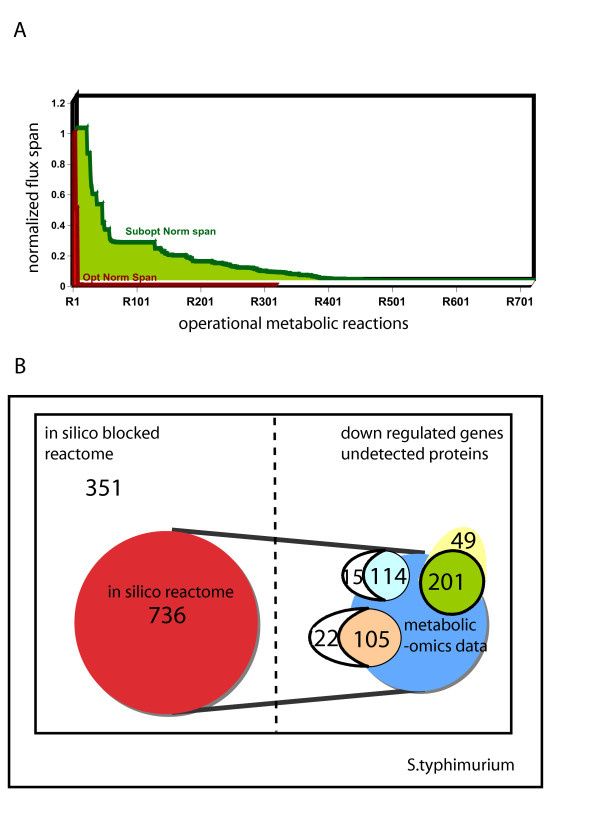
**Metabolic capacity of *S. typhimurium *in the host cell or *in vivo***. (A) *In silico *flux range for operational metabolic reactions in a simulated host environment using flux variability analysis. The plot shows the flux span normalized to the maximum cytochrome b556 production flux. The red area indicates the flux span during optimal growth of salmonella, while the yellow area represents the flux span during suboptimal growth. During sub-optimal growth, a higher internal flexibility is observed as compared to that during optimal growth. 417 reactions are predicted to be operational during optimal growth and an additional 319 are operational during suboptimal of salmonella inside the host cell. (B) Comparison of *in silico *prediction of the reactome (red) to the established metabolic proteome (light blue) [5] and transcriptome (green) [2] of *S. typhimurium *extracted from macrophage post infection and under different in vitro environmental conditions (orange) [56]; 88% of the metabolic proteome, 80% of the metabolic transcriptome and 83% of the proteome established as a result of 3 different environmental responses *in vitro *are a subset of *the in silico *predicted reactome. Several genes down regulated in the metabolic transcriptome are associated with blocked reactions (i.e. inactive reaction in host cell environment) and correlate with those predicted *in silico *using FVA.

*In silico *prediction of metabolic pathways utilized during infection allows us to identify redundant metabolism that the pathogen could exploit in order to survive and replicate. During conditions of infection it is reasonable to assume that *Salmonella *cells are under an energy limitation. Hence, ATP generated is fully coupled to biosynthesis and growth [55]. Given the possible nutrients provided in the host-cell environment, we used flux variability analysis (FVA) to identify which reactions can be utilized [56] and found that 417 reactions span a large range of flux values (Figure 5A) when biomass production is optimal. These 417 reactions (see Additional file 3) encompass all the possible reactions (reactome) that could be used by *Salmonella *during infection. When the biomass production is allowed to be suboptimal (ie. values less than the maximum biomass production), an additional 319 reactions (736 in total) could be used by *Salmonella *in the host cell environment (see Additional file 3). The constraining conditions of the simulation (listed as media composition in Additional file 6), were derived by literature mining to identify a media composition that could closely mimic nutrient availability for *S. typhimurium *in the host cell. We also used FVA to identify blocked reactions and thus pathways that will never be used by the pathogen under the described conditions *in vivo *during infection. These reactions are typically those catalyzed by proteins that cannot be detected in the proteome or those genes that are down regulated under similar conditions. Our model simulation results of these blocked reactions (see Additional file 3) along with our gene expression data identify correctly fatty acid degradation metabolic genes (complete fad operon) to be non-operational during the above conditions. Thus, there is some correlation between blocked reactions found by FVA and unexpressed genes during infection.

We compiled the list of all proteins that comprise the *in silico *reactome and compared that to previously determined proteome of *S. typhimurium *extracted from macrophage cells after infection (Figure 5B). Of the 315 proteins identified with a high level of confidence in *S. typhimurium *[5,57], 129 have an annotated metabolic function (metabolic proteome) and in our model *i*RR1083. Eighty of these proteins were associated with reactions present in the reactome calculated by FVA analysis during optimal growth, while only 34 were identified in the host-cell reactome when growth was sub-optimal. In another experiment, where the proteome of *S. typhimurium *was probed under different *in vitro *environmental conditions, a total of 507 proteins were identified [56], of which 127 were accounted for in our metabolic reconstruction. Out of these 127 proteins, 83 and 22 were associated with the optimal and suboptimal host cell reactome, respectively, as calculated using FVA. Most of the proteins that were found in both proteomic data sets and associated with blocked reactions, as calculated by FVA, were tRNA synthetases (7 out of 15 [5] and 13 out of 22 [56]). Incorporating charged tRNAS instead of free amino acids in the biomass reaction would change the tRNA synthetase reactions from blocked to part of the optimal reactome and would result in higher agreement between the optimal reactome and identified proteins.

Using a similar strategy we compared micro-array gene expression data obtained from *S. typhimurium *extracted from macrophage cells [2]. Of the 919 genes that were differentially expressed, 250 genes had a metabolic function, thus comprising the metabolic transcriptome. When we compared those with the gene set represented by our FVA analysis reactome, we identified 80% of the genes in the metabolic transcriptome were associated with reactions in the reactome. Of the remaining 49 that were not present in our *in silico *reactome, 65% of the genes were down-regulated and formed a subset of the blocked reactions from our FVA analysis (Figure 5B). Although the accuracy of prediction is very good, it could be increased by improving our knowledge of existing nutrients in the macrophage or incorporating regulation into the model. By identifying preferred metabolic pathways we can better understand how the pathogen responds to its microenvironment during invasion and infection.

## Discussion

This study describes a genome-scale metabolic model, *i*RR1083, of the Gram-negative intracellular pathogen *S. typhimurium*. The model has been reconstructed by integration of existing genomic, proteomic, and phenotypic experimental data. Comparison between model predictions and experimental data demonstrated an accuracy of more than 80% for growth and virulence phenotypes. The model allowed us to use flux balance analysis to analyze growth and virulence genes given the environment the intracellular pathogen encounters. Further, gene expression experiments and computational analysis allowed us to identify metabolic enzymes and pathways that are potentially active during infection. Model-development is an iterative process that moves from a genome annotation and biochemistry of the organism to the development of a genome-scale metabolic network that can be used as a fully functional *in silico *organism to compute physiological phenotypes. Our model iRR1083 serves also as a framework to integrate further gene expression and proteomic data and explain the expression level of specific gene products during infection.

Computing cellular functions from reconstructed networks allowed us to understand the relationship between environmental factors (nutrient availability) and genetic factors (mutations or deletions) and their integrated function to orchestrate cell phenotypes (virulence or attenuation). Knowledge of the functional status of metabolic genes under defined constraints allows us to interpret the role of critical nodes in a cellular metabolic network. The function of redundant pathways under internally perturbed conditions, such as in the host cell during infection, provides important insights into metabolic network structure. Although this internal robustness has been experimentally demonstrated, building a complete catalog of genes essential for growth under a myriad of conditions for any organism is time-consuming and laborious. A computational approach that predicts gene essentiality with high accuracy is thus of great value. Model predictions of virulence for different mutants improved from 80% to 91% upon altering host-cell environment components (by adding glutamine, and removing thymidine, guanosine, adenosine, and deoxyadenosine) and this shows how the model can be used as a tool for providing insights into the role of metabolism in infection and pathogenesis. To date, constraint-based models cannot predict effects of toxic intermediates and methods are needed that allow for prediction of concentrations within constraint-based models.

A biomass reaction (representing the conversion of biomass components into one gram dry weight of biomass) is needed to accurately predict the effects of gene deletions on growth and viability. Although predictions have been made in the past with approximations of the biomass function to that of *E. coli*, there are some major differences in fatty acid and amino acid composition that require experimental measurements to determine the drain of different metabolites and the cost of their production on the function of the organism. Our study has addressed this issue by directly determining the components of *Salmonella *biomass, thus increasing the validity and predictive accuracy of model iRR1083.

*S. typhimurium *is a pathogen that is the leading cause of human gastroenteritis and is used as a mouse model for human typhoid fever. Although more than 2000 serovars exist, the LT2 strain, whose genome sequence has been used here for building model iRR1083, is one of the principal strains for cellular and molecular biology of *Salmonella*. Attenuated mutants have been used not only as live oral vaccines against *Salmonella *infection but also to express antigens from other pathogens and to deliver proteins to solid tumors. One reason for attenuation or avirulence is the deletion or functional silencing of a genetic element in the bacterium resulting in the impairment of the intracellular survival capacity of *S. typhimurium*. Our *in silico *analysis shows the ability to predict such effects with good accuracy. At this point the host cell metabolic environment has been inferred indirectly from gene-expression data. Improved accuracy in directly determining what metabolites are available in the host cell environment and incorporation of transcriptional regulatory rules into *i*RR1083 will allow a more precise analysis of metabolic selective pressures a pathogen encounters during infection.

In addition to pathogenicity and virulence analysis, the *S. typhimurium *metabolic reconstruction can be a valuable tool to interpret several 'omics' data types. Although there is not a direct correlation between gene or protein expression levels and metabolic fluxes, the genome-scale model serves as a platform to visualize these large data sets and interpret them in the context of operational, redundant or incomplete metabolic pathways. This provides a better understanding of cellular phenotypes and offers insights into how system level properties affect survival or sensitivity of pathogens to factors in the environment provided by the host cell. For example, the model prediction of *fad *genes not being operational during early stages of infection (optimal growth) and operational during sub-optimal growth can be explained by the fact that fatty acids are not utilized in the initial stages of infection by *S. typhimurium *[45]. This observation along with the calculated host cell reactome from *i*RR1083 could suggest a switch from optimal to sub-optimal growth or a change in nutrients available for *Salmonella *inside host cell tissue. This switch is concomitant with a marked decrease in growth during persistence or chronic infection. Growth rates are presumably higher (optimal) during the initial stage of infection. The entire reactome of *Salmonella *as predicted by the model provides many hypotheses that need to be further investigated to identify the implications of metabolic pathways and functions in pathogenesis. Metabolomic data have become more readily available and easier to measure. They can be used to apply additional constraints to the model [58], thus improving model predictions of cell function and global phenotypes.

The genome-scale model of *S. typhimurium *we have developed here also presents us with the opportunity to interrogate several other serovars or species that belong to the *Salmonella *genus. For example, *panD *is the only gene that is non-functional in *Salmonella typhi*, and is predicted to be required for virulence in specific conditions. The presence of pseudogenes in other serovars can be analyzed in the context of the genome-scale model to present us with systemic similarities and differences that may or may not play a role in the pathogenic potential of all the four species. Serovars lacking *panD *would require panthothenate from its host. Thus, compounds that affect pantothenate transport could potentially be used as antibiotics. Such analyses across different serovars require genome-scale models and might accelerate the identification of rational drug targets.

Although many of the reactions in the *i*RR1083 model for *S. typhimurium *are conserved in models for *E. coli *MG1655, there are unique metabolic and genetic differences that affect growth phenotypes in response to different environmental or genetic perturbations. The two organisms are each capable of utilizing unique carbon sources, which are related to differences in metabolic networks (unique transporters or enzymes) or differences in regulation or kinetics that affect flow through shared reactions common to both organisms (reactions are present to catabolize carbon sources in both metabolic networks but one organism does not use them as sole carbon sources). We also found that there are differences in the number of isozymes or alternative reactions/pathways that enable one organism to be more robust against gene deletions in a glucose minimal media condition. We anticipate that comparisons of model-predicted essential genes in more environmental conditions will lead to the identification of more metabolic differences between the two organisms, as the analysis was only performed for aerobic growth in glucose minimal media. These metabolic differences might shed light onto the evolutionary differences between the two organisms and the nutrients they are or were exposed to.

The genome-scale metabolic network reconstruction presented here supports the notion of a robust minimal set of pathways that *Salmonella *requires for intracellular survival during infection [52]. Our model allows *in silico *simulation of different nutritional requirements and constraints as they may be present in different host cell types or tissues that are difficult to directly address experimentally. Our model can generate hypotheses that can be validated by using mutant strains, high-throughput data, and selective analysis of pathways. Such an endeavor has the potential to lead to an improved understanding of how intracellular pathogens utilize the existing microenvironment of the host. This in turn might lead to the identification of conserved metabolic pathways that can be targeted by novel interfering compounds.

## Conclusion

We have presented here extensive datasets within a reconstruction of a genome-scale metabolic model for *S. typhimurium *(iRR1083). This model has been validated by experimental data and by legacy data. It demonstrates an accuracy of 80% for growth and virulence phenotypes. Thus the model iRR1083 provides a suitable platform for computing cellular phenotypes for *Salmonella *and for further integration of high-throughput data.

Biomass composition of *Salmonella *was determined experimentally, which increased the accuracy of our model building and also showed distinctive difference to that of *E. coli*. Constraint-based analysis using the metabolic reconstructions iRR1083 for *S. typhimurium *and MG1655 for *E. coli *identified unique metabolic differences between these two bacteria.

Expression profiling of selective key metabolic enzymes in *Salmonella *during infection of macrophages identified operational and non-operational metabolic pathways. Flux variabity analysis of metabolic pathways identified a minimal set of reactions required for *Salmonella *intracellular survival.

This study thus provides another high-resolution reconstruction of microbial metabolism of an intracellular pathogen next to that of *M. tuberculosis *and demonstrates its suitability for analyzing bacterial metabolic networks during infection. From further validation and testing of hypotheses generated from these reconstruction efforts we expect to arrive at a conserved set of metabolic reactions that interdigitate with those of the host cell and might thus provide novel targets for therapeutic intervention.

## Methods

### Metabolic Network Reconstruction

The reconstruction process for *S. typhimurium *is outlined schematically in the Additional file 9. The process followed the approach outlined by Edwards and Palsson (Edwards and Palsson, 2000). The reconstruction was carried out in SimPheny™ (Genomatica, Inc., San Diego, CA), a platform for development of cellular models. Lists of genes, proteins and reactions, metabolites and exchange fluxes that are included in the resulting final reconstruction can be found in the Additional file 1.

The initial draft reconstruction was built from the annotated genome [25] of *Salmonella enterica *serovar *typhimurium *LT2 (*S. typhimurium LT2*), which is available at TIGR. Genes to proteins to reaction associations (GPR) were confirmed by comparing homology of *S. typhimurium *gene sequences to *E. coli *gene sequences and biological information databases such as EXPASY, KEGG, and BioCyc. These GPR associations connect genetic data to reactions in the metabolic network [26] and allow for subsequent evaluation of how genetic perturbations affect metabolic phenotypes.

### Biomass Composition and Reaction

A reaction that represents biomass production is included in the model to account for the drain of precursors and macromolecular building blocks into biomass. Biomass was represented as a linear combination of all the macromolecular components (lipid, glycogen, lipopolysaccharide, and peptidoglycan) or monomers of macromolecules (amino acids and nucleotides). Amino acid and fatty acid composition were experimentally measured using standard methods described in manual of bacteriological methods [59]. The relative fatty acid composition was used to specify the average fatty acid content on the phospholipds and diacylglyerol. *S. typhimurium *LT2 data from the literature was then used to determine the amount of lipids (and their phospholipid composition), lipopolysaccharide, peptidoglycan, and glycogen. The growth associated ATP maintenance and soluble metabolite pools were assumed to be the same as reported for *E. coli *[9]. Complete details of the cellular composition and references used for the *S. typhimurium *biomass reaction are provided in the Additional file 2. The biomass reaction thus represents the weighted combination of components forming the dry weight of the cell and the amount of ATP hydrolysis, needed for energy during growth and cellular maintenance.

### Flux Balance Analysis

Fluxes through metabolic reactions across the network can be calculated using flux balance analysis (FBA) [13]. With FBA, the biological system is assumed to be at steady-state and so all intracellular metabolite concentrations and fluxes are assumed to be constant. The steady-state assumption makes it possible to compare the simulation results directly to data obtained from cells growing at a fixed growth rate. FBA is formulated as an optimization problem, where constraints are imposed that limit flux values (steady-state mass balance constraint and upper and lower bounds for fluxes based on thermodynamics and substrate uptake constraints). These constraints define the range of values that fluxes can take. An objective function is also used to compare flux distributions (**v**) that satisfy all the constraints in order to find optimal flux distributions. Flux through the biomass production reaction has successfully been used as an objective function for *E. coli *(along with other objective functions [60,61], and was used for FBA performed in this study. Thus, FBA predicts an optimal growth yield and a flux distribution(s), which correspond to this maximal growth yield. Alternate optimal solutions exist, and flux variability analysis [62] was additionally used to identify the ranges individual fluxes can take while still achieving the maximal growth yield.

### *In silico *constraints and media composition

Three different environments were used to provide constraints for simulations: (i) M9 minimal medium (contains salts, phosphate, sulfate, ammonium minerals) with an added carbon source; (ii) LB-media (composition was based on a typical analysis of the yeast extract and tryptone provide by the manufacturer); (iii) host-cell nutrient environment (representing nutrient conditions inside a host-cell during Salmonella infection). While the host-cell nutrient environment was difficult to ascertain, it was based on an extensive literature review to identify a possible composition. Details of all simulated media conditions are provided in the Additional file 6. For simulation of aerobic growth, the following external metabolites were allowed to freely enter and leave the network: phosphate, ammonium, sulfate, water, oxygen, and proton. All metabolites that were not media components (potential products) were only allowed to leave the system.

Validation of the model against Biolog data for *S. typhimurium *LT2 (available from Biolog website, ) was performed by optimizing biomass production with different carbon and nitrogen sources. A maximum uptake rate of 5 mmol per gram of dry weight per hour (mmol/gDW cell/h) was used for the different carbon and nitrogen sources. Different nitrogen sources were simulated with pyruvate as a carbon source and different carbon sources were simulated with ammonium as a nitrogen source.

### Gene Essentiality

Genetic changes, such as gene deletions and mutations, can be simulated by adding additional constraints to individual fluxes [38]. For example, deletion of an enzyme subunit would force flux values through associated reactions to be zero, unless other isozymes are present. The essentiality of a given gene was determined by calculating the maximal growth rate using FBA when fluxes through associated reactions were constrained to be zero. This type of analysis identifies lethal gene deletions (where maximum growth rate is zero) and non-lethal gene deletions (where maximum growth rate is greater than zero). Gene essentiality is dependent on media composition, and all three growth environments (M9 minimal media with glucose, LB media, and host-cell nutrient environment) were analyzed separately.

### Growth and Infection Experiments

All growth experiments were carried out using the strain *S. enterica serovar typhimurium *LT2. Aerobic batch cultivation was done in an incubator shaker at 37°C and 100 rpm. Minimal M9 medium was supplemented with 6 different carbon sources (glucose, fructose, glycerol, arabinose, ribose and xylose). The cell culture optical density (OD) was monitored at regular intervals using a Beckman DU spectrophotometer at 600 nm. The OD was converted into g dry weight using a previously determined biomass calibration standard curve using the same spectrophotometer (gDW = 0.7396·OD_600_). Samples were also withdrawn every 2 hours, filtered and the supernatants were frozen and stored at -80°C for HPLC analysis to analyze substrate utilization and product formation profiles.

### HPLC Analysis

Organic acid and sugar analysis was done using an ion exchange Aminex 87H^+ ^column (Biorad). A 5 mM Sulfuric acid mobile phase at a flow rate of 0.5 ml/min at 65°C was used for separation. Both UV and RI (refractive index) detector were employed. Small molecules were quantified using a previously determined calibration curve.

### Quantitative Gene expression using GeXP™

Infection of the macrophage like cell line (RAW246.7) was carried out as described [63]. RNA was purified from harvested bacterial cells using the protocol from RNeasy Kit (QIAGEN) and quantitated using UV detection at 260 nm and 280 nm. The GenomeLab™ GeXP Genetic Analysis System (Beckman) was used for quantitative gene expression analysis of the selected metabolic transcriptome of *S. typhimurum*. GeXP employs eXpress Profiling (XP-PCR), a patented technology for multiplex gene expression profiling analysis by which up to 30 genes can be easily multiplexed in the same reaction. We used a protocol that comes with GeXP Reagent kits that involved five basic steps: 1) Primer design; 2) cDNAsynthesis; 3) PCR; 4) Separation on the GenomeLab GeXP Genetic Analysis System; 5) Fragment Analysis and Expression Profiling. The data was then analyzed using the GeXP analysis proprietary software (Beckman, Inc.), Excel (Microsoft, Inc.) and Matlab (Mathworks, Inc.). Primers were designed using proprietary software provided by Beckman (see Additional file 10).

## Authors' contributions

AR and JR conducted the reconstruction of *Salmonella typhimurium i*RR1083 and performed all the analyses. AR and SS obtained all the experimental data. AR and SD planned and designed the study, analyzed, and interpreted all the data. AR, JR, and SD wrote the manuscript. BP provided critical advice and suggestions. All authors approve the content of this manuscript.

## Supplementary Material

Additional file 1 ***Salmonella *metabolic network *i*RR1083**. Gene-Protein-Reaction associations in the final version of the reconstruction *i*RR1083. Click here for file

Additional file 2 **Cellular composition of *S. typhimurium***. Experimentally determined values for Salmonella cellular content of amino acids, carbohydrates, DNA, RNA, selected organic and inorganic compounds, and fatty acid composition of membrane and cell wall components. Click here for file

Additional file 3 **Flux Variability Analysis**. *In silico *reactome as determined by flux variability analysis (FVA). Click here for file

Additional file 4 **Comparison of qualitative phenotyping (experimental vs in silico)**. Predictions for 163 possible carbon sources and 98 possible nitrogen sources were compared with experimental data. Click here for file

Additional file 5 **Gene essentiality comparison between *E. coli *and *S. typhimurium***. List of discrepancies in gene essentiality predictions between orthologs which are present in genome-scale models of *S. typhimurium *(iRR1083) and *E. coli *(iAF1261). Click here for file

Additional file 6 **Media compositions used *in silico *for M9, LB and host cell environment**. Carbohydrates, amino acids, vitamins, minerals, inorganic molecules, nucleotides, and amines used for *in silico *analysis. Click here for file

Additional file 7 ***Salmonella *serovars *typhimurium, typh*i, *paratyphi*, and *enteritidis***. List of genes present in serovar *typhimurium *and absent or non functional (pseudogenes) in serovars *typh*i, *paratyphi *and *enteritidis*. Click here for file

Additional file 8 **Selected metabolic gene expression during infection of macrophages by *Salmonella***. Selected metabolic genes used for measuring gene expression or mRNA transcript levels in *S. typhimurium *extracted from macrophages post infection. Click here for file

Additional file 9 **Reconstruction of *Salmonella *metabolic network**. Flow chart for reiterative model building for *Salmonella *network *i*RR1083. Click here for file

Additional file 10 **Primer Sequences**. Primer Sequences used to measure gene expression or mRNA transcript levels of the chosen metabolic gene set described in S6 using GEXP. Click here for file
