# Supplementary material for: Constraint-based analysis of metabolic capacity of Salmonella typhimurium during host-pathogen interaction
Source: BMC Syst Biol. 2009 Apr 8;3:38. doi: 10.1186/1752-0509-3-38 (PMC2678070; doi:10.1186/1752-0509-3-38)
Supplement: Additional file 6 — Media compositions used in silico for M9, LB and host cell environment. Carbohydrates, amino acids, vitamins, minerals, inorganic molecules, nucleotides, and amines used for in silico analysis. [file 1752-0509-3-38-S6.doc]

*Supplemental Data S4: Media compositions used in silico for M9, LB and host cell environments for simulations.*

| **Component** | **M9 medium**^a^ | ***In silico* LB medium**^b^ | ***In silico***  **Host cell Environment**^c^ |
| --- | --- | --- | --- |
| **Carbohydrates** |  |  |  |
| Glucose | + | + | + |
| Fructose |  |  | + |
| Fucose |  |  | + |
| galactitol |  |  | + |
| galactonate |  |  | + |
| Galactose |  |  | + |
| Glucarate |  |  | + |
| Galacatarate |  |  | + |
| gluconaate |  |  | + |
| glucuronate |  |  | + |
| L-arabinose |  |  | + |
| maltose |  |  | + |
| mannitol |  |  | + |
| mannose |  |  | + |
| melibiose |  |  | + |
| N-acetylglucosamine |  |  | + |
| N-acetylneuraminic acid |  |  | + |
| rhamnose |  |  | + |
| ribose |  |  | + |
| sorbitol |  |  | + |
| cellobiose |  |  | + |
| 1,2 propanediol |  |  | + |
|  |  |  |  |
| **Amino acids** |  |  |  |
| Alanine |  | + | + |
| Arginine |  | + | + |
| Aspartic acid |  | + |  |
| Asparagine |  |  | + |
| Cysteine |  |  | + |
| Cystine |  | +^d^ |  |
| Glutamic acid |  | + | + |
| Glutamine |  |  |  |
| Glycine |  | + |  |
| Histidine |  | + | + |
| Isoleucine |  | + | + |
| Leucine |  | + | + |
| Lysine |  | + | + |
| Methionine |  | + | + |
| Proline |  | + | + |
| Threonine |  | + | + |
| Tryosine |  | + |  |
| Phenylalanine |  | + |  |
| Serine |  | + |  |
| Tryptophan |  | + |  |
| Valine |  | + | + |
|  |  |  |  |
| **Vitamins** |  |  |  |
| Thiamine (B1) |  |  | + |
| Riboflavin (B2) |  | +^d^ |  |
| Calcium pantothenate (B5) |  | + | + |
| Folic acid (B9) |  | +^d^ |  |
| Niacin (PP) |  | +^d^ |  |
|  |  |  |  |
| **Minerals & Inorganic Molecules** |  |  |  |
| Sodium | + | + | + |
| Chloride | + |  | + |
| Sulfate | + | + | + |
| Potassium | + | + | + |
| Phosphate | + | + | + |
| Calcium | +^d^ | +^d^ | +^d^ |
| Magnesium | + | + | + |
| Selenium |  | +^d^ |  |
| Zinc |  | +^d^ |  |
| Arsenic |  | +^d^ |  |
| Cadmium |  | +^d^ |  |
| Mercury |  | +^d^ |  |
| Ammonium | + |  |  |
| DMSO |  |  | + |
| H^+^ | + | + | + |
| H_2_O | + | + | + |
| O_2_ | + | + | + |
| Nitrate |  |  | + |
| Sulfite |  |  | + |
|  |  |  |  |
| **Nucleotides/nucleosides** |  |  |  |
| Inosine |  | + | + |
| Hypoxanthine |  | + | + |
| Deoxycytidine |  | + | + |
| Thymidine |  | + | + |
| Uracil |  | + | + |
| Uridine |  | + | + |
| Deoxyadenosine |  | + | + |
| Adenosine |  | + | + |
| Guanosine |  |  | + |
| Cytosine |  |  | + |
|  |  |  |  |
| **Amines** |  |  |  |
| Allantoin |  |  | + |
| Carnitine |  |  | + |
| Polyamine |  |  | + |
| Ethanolamine |  |  | + |

^a^ Based on minimal media used in experiments

^b^ Based on a manufacturer composition of yeast extract, tryptone from DIFCO

^c^ Based on literature review to identify probable compounds in the host cell

^d^ No exchange reaction for this compound is included in the model, so simulations do not account for the uptake of the compound.
