# Supplementary material for: Constraint-based analysis of metabolic capacity of Salmonella typhimurium during host-pathogen interaction
Source: BMC Syst Biol. 2009 Apr 8;3:38. doi: 10.1186/1752-0509-3-38 (PMC2678070; doi:10.1186/1752-0509-3-38)
Supplement: Additional file 8 — Selected metabolic gene expression during infection of macrophages by Salmonella. Selected metabolic genes used for measuring gene expression or mRNA transcript levels in S. typhimurium extracted from macrophages post infection. [file 1752-0509-3-38-S8.doc]

Supplemental Data S6: Selected metabolic gene set chosen for gene expression or mRNA transcript level detection in *S. typhimurium* extracted from macrophages post infection using GEXP.

| **GENE LOCUS ID** | **ABBRV** | **PROTEIN FUNCTION** |
| --- | --- | --- |
|  | | |
| **CARBOHYDRATE METABOLISM** | | |
| STM0169 | *gcd* | Glucose dehydrogenase |
| STM4221 | *pgi* | glucose-6-phosphate isomerase |
| STM2403 | *glk* | Glucokinase |
| STM1886 | *zwf* | Glucose-6-phosphate 1-dehydrogenase |
| STM3542 | *gntK* | Gluconate kinase 2 |
| STM2081 | *gnd* | 6-phosphogluconate dehydrogenase |
| STM1885 | *edd* | 6-phosphogluconate dehydratase |
| STM4062 | *pfkA* | Phosphofructokinase |
| STM1326 | *pfkB* | 6-phosphofructokinase II |
| STM3068 | *fba* | Fructose-bisphosphate aldolase |
| STM4415 | *fbp* | Fructose-bisphosphatase |
| STM3069 | *pgk* | Phosphoglycerate kinase |
| STM1349 | *pps* | Phosphoenolpyruvate synthase |
| STM1378 | *pykF* | Pyruvate kinase I |
| STM1888 | *pykA* | Pyruvate kinase II |
| STM3939 | *cyaA* | Adenylate cyclase |
|  | | |
| **FATTY ACID METABOLISM** | | |
| STM0309 | *fadE* | Acyl-coenzyme A dehydrogenase |
| STM3982 | *fadA* | 3-ketoacyl-CoA thiolase |
| STM1818 | *fadD* | Long-chain-fatty-acid--CoA ligase |
| STM4184 | *aceA* | Isocitrate lyase |
| STM1238 | *icdA* | Isocitrate dehydrogenase |
| STM0730 | *gltA* | Citrate synthase |
| STM3359 | *mdh* | Malate Dehydrogenase |
|  |  | isopropyl Malate Dehydrogenase |
| STM1566 | *sfcA* | NAD-dependent malic enzyme |
| STM2472 | *maeB* | NADP-dependent malic enzyme |
| STM3500 | *pckA* | Phosphoenolpyruvate carboxykinase |
| STM4119 | *ppc* | Phosphoenolpyruvate carboxylase |
| STM4183 | *aceB* | Malate synthase |
| STM2309 | *menD* | 2-oxoglutarate decarboxylase |
| PSLT002 | *PSLT002* | Putative phospholipase D |
| STM3961 | *pldB* | Lysophospholipase L |
|  |  |  |
| **ACID RESISTANCE – pH METABOLISM** | | |
| STM0234 | *ldcC* | Lysine decarboxylase 2 |
| STM2559 | *cadA* | Lysine decarboxylase, induciblle |
| STM2558 | *cadB* | APC family lysine/cadaverine transport protein |
| STM4296 | *adi* | Arginine decarboxylase |
| STM4294 | *adiC* | Arginine/agmatine antiporter |
| STM3086 | *adc* | Biosynthetic arginine decarboxylase |
| STM0701 | *speF* | Ornithine decarboxylase isozyme, inducible |
| STM3114 | *speC* | Ornithine decarboxylase isozyme |
| STM0700 | *potE* | putrescine/ornithine antiporter |
| STM2793 | *ganP* | gamma-aminobutyrate transport |
|  |  | Glutamate decarboxylase |
|  | | |
| **AMINO ACID METABOLISM** | | |
| STM0978 | *aroA* | 5-enolpyruvylshikimate-3-phosphate synthase |
| STM1724 | *trpD* | anthranilate phosphoribosyltransferase / anthranilate synthase component II |
| STM1723 | *trpE* | anthranilate synthase, component I |
| STM2384 | *aroC* | chorismate synthase |
| STM3903 | *ilvE* | branched-chain amino-acid aminotransferase |
| STM4007 | *glna* | glutamine synthetase |
| STM1310 | *nadE* | NAD synthetase |
| STM2362 | *purF* | amidophosphoribosyltransferase |
| STM2072 | *hisD* | histidinol dehydrogenase |
| STM2075 | *hisH* | glutamine amidotransferase |
| STM2071 | *hisG* | ATP phosphoribosyltransferase |
| STM2073 | *hisC* | histidinol phosphate aminotransferase |
| STM0603 | *ybdL* | putative aminotransferase |
| STM2667 | *pheA* | Chorismate mutase |
|  | | |
| **OXIDATIVE PHOSPHORYLATION** | | |
| STM0443 | *cyoA* | cytochrome o ubiquinol oxidase subunit II |
| STM0439 | *cyoE* | protohaeme IX farnesyltransferase |
| STM0732 | *sdhC* | succinate dehydrogenase, cytochrome b556 |
| STM2328 | *nuoA* | NADH dehydrogenase I chain A |
| STM4343 | *frdA* | fumarate reductase, anaerobic |
| STM0360 |  | cytochrome BD2 subunit I |
| STM3867 | *atpA* | membrane-bound ATP synthase, F1 sector, alpha-subunit |
| STM3864 | *atpC* | membrane-bound ATP synthase |
| STM1211 | *ndh* | respiratory NADH dehydrogenase 2; cupric reductase |
| STM2501 | *ppk* | polyphosphate kinase |
| STM0483 | *apt* | adenine phosphoribosyltransferase |
|  | | |
| **EFFLUX TRANSPORTERS** | | |
| STM0942 | *ybjZ* | MACROLIDE EFFLUX |
| STM0460 | *mdlA* | putative ABC superfamily (atp) transporter |
| STM0461 | *mdlb* | putative ABC superfamily (atp) transporter |
| STM2263 | *yojI* | putative ABC-type multidrug/protein/lipid transport system, |
| STM0173 | *yadH* | antibiotic transport system permease protein |
| STM0172 | *yadG* | ABC-type multidrug transport system |
|  |  |  |
| **QUINONES** |  |  |
| STM4233 | *ubiC* | chorismate pyruvate lyase |
| STM2306 | *menC* | O-succinylbenzoate-CoA synthase |
|  |  | menaquinone biosynthesis methyltransferase |
|  | | |
| **REACTIVE OXYGEN SPECIES METABOLISM** | | |
| STM4106 | *katG* | Peroxidase/catalase |
| STM1341 | *btuE* | Glutathione peroxidase |
| STM2818 | *gshA* | gamma-glutamate-cysteine ligase |
| STM1451 | *gst* | glutathionine S-transferase |
| STM3551 | *ggt* | gamma-glutamyltranspeptidase |
