# Supplementary material for: Constraint-based analysis of metabolic capacity of Salmonella typhimurium during host-pathogen interaction
Source: BMC Syst Biol. 2009 Apr 8;3:38. doi: 10.1186/1752-0509-3-38 (PMC2678070; doi:10.1186/1752-0509-3-38)
Supplement: Additional file 9 — Reconstruction of Salmonella metabolic network. Flow chart for reiterative model building for Salmonella network iRR1083. [file 1752-0509-3-38-S9.ppt]

## Slide 1
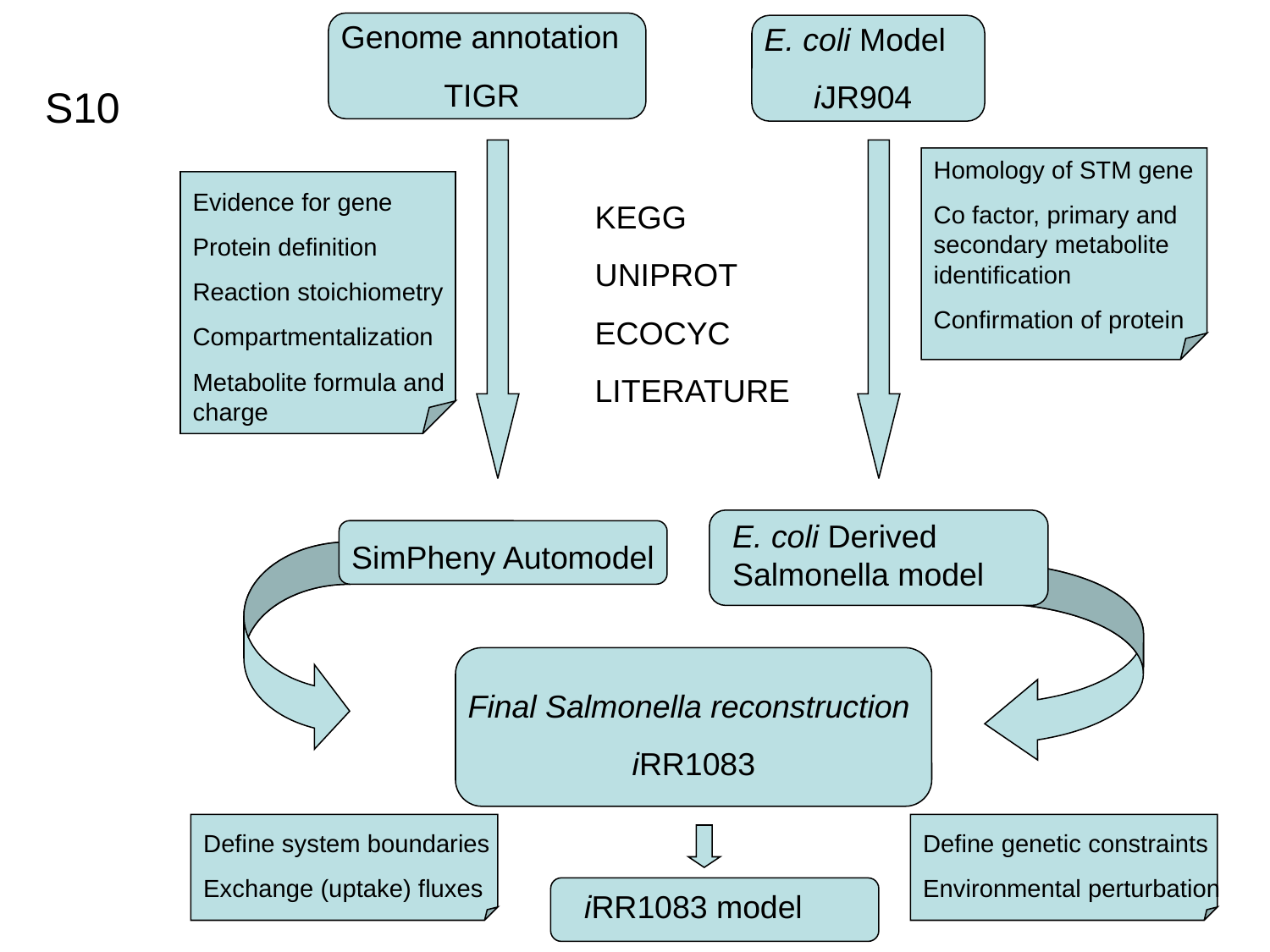

Genome annotation
TIGR
E. coli Model
iJR904
S10
Homology of STM gene
Co factor, primary and secondary metabolite identification
Confirmation of protein
Evidence for gene
Protein definition
Reaction stoichiometry
Compartmentalization
Metabolite formula and charge
KEGG
UNIPROT
ECOCYC
LITERATURE
E. coli Derived Salmonella model
SimPheny Automodel
Final Salmonella reconstruction
iRR1083
Define system boundaries
Exchange (uptake) fluxes
Define genetic constraints
Environmental perturbation
iRR1083 model
